# Informal care and gifts to and from older people in Europe: The interlinks between giving and receiving

**DOI:** 10.1186/s12913-016-1830-7

**Published:** 2016-10-21

**Authors:** Florian Tomini, Wim Groot, Sonila M. Tomini

**Affiliations:** 1 Top Institute for Evidence-Based Education Research (TIER), Maastricht University, Maastricht, Netherlands; 2 Amsterdam School of Economics, University of Amsterdam, Amsterdam, Netherlands; 3 Department of Health Services Research, Faculty of Health, Medicine and Life Sciences, Maastricht University, Maastricht, Netherlands; 4 Maastricht Graduate School of Governance and United Nation University-Merit, UNU-Merit, Maastricht University, Maastricht, Netherlands; 5 Department of Economics, University of Liege, Liège, Belgium

**Keywords:** Informal care, Informal gits, Older people, Europe, SHARE data

## Abstract

**Background:**

Transfers of money and help with daily activities by family and friends are important sources of support for older people and contribute to their well being. On the other hand, older adults are not only recipients of support but also important providers of support and financial transfers as giving and receiving are often reciprocal. For this, it is important to understand the determinants of receiving and giving money and help as well as the relationship between these two.

**Methods:**

The aim of this paper is to explore the relationship between giving and receiving of the same or of different types of transfers as well as to get more insights in the motivation behind giving and receiving of money gifts or informal care. We use data from the Survey of Health Aging and Retirement in Europe and employ a multinomial logit model to analyse 16 different categories resulting from combining information on the incidence of giving and receiving of both informal care and financial gifts.

**Results:**

We show that despite the differences that exist in the incidence of giving and receiving of both informal care and financial gifts there are clearly a few patterns that are consistent between the European countries in our analysis. Both ‘altruistic-like’ and ‘exchange-like’ motives are more likely to increase by age, gender and physical proximity of network members, while ‘reciprocal-like’ giving and receiving is more likely among females and those with a network at close distance.

**Conclusions:**

Our results show that the incidence of informal care and gifts to and from older people is related to particular characteristics and transfers patterns. Further research should be dedicated to exploring the situations leading to the ‘altruistic-like’ and ‘exchange-like’ combinations of transfers.

**Electronic supplementary material:**

The online version of this article (doi:10.1186/s12913-016-1830-7) contains supplementary material, which is available to authorized users.

## Background

With an ageing society the provision of care and support to older people who are less able to look after themselves has become an issue of concern [1–6]. With the increasing pressure on long-term care expenditures, informal care delivered by family and friends is seen as one of the main factors to ensure the sustainability of care systems [7, 8]. Previous research has shown that informal care may contribute to reducing the growth in total care expenditures as it may reduce the use of formal care and delay admission to nursing homes [3, 9, 10]. However, formal and informal care are not always perfect substitutes to each-other [11] and often support provided by family members complements rather than substitutes for formal arrangements [12]. Moreover, informal solidarity arrangements are frequently based on the notion of reciprocity and mutual giving and receiving: you give care now in the expectation that you will receive care back when needed [13, 14]. It is also recognized that older people are frequently not only the recipients of support but also important support providers [15]. For this reason, receiving and giving of the informal care is often correlated. The same can be said for financial transfers, people who are likely to give may also be more likely to receive and vice versa [15, 16]. Given the complex nature of informal support transfers between family members and older people, it is important to understand the main patterns and determinants of receiving and giving as well as the motivation behind such transfers.

This paper aims to increase our knowledge and insight on informal solidarity arrangements and the patterns of transfers as well as the motivation behind giving and receiving of monetary transfers from and to people older than 50 years old in Europe. We explore who gives and who receives informal care and who gives and receives monetary transfers in Europe and how these transfers are related to each other. Moreover, we are interested in exploring the links between giving and receiving of the same or of different types of transfers. For instance, are those who receive informal care or money more likely to also provide them to others. And, if not, what types of transfers are provided together, and what transfers are provided in return? We account for all the potential combinations that exist between the receiving and giving and also account for the differences between different European countries (given the different formal solidarity arrangements that exist in each of these countries).

### Theoretical considerations behind helping others

The complexity of the motivations behind informal gifts and care to family members has attracted the attention of researchers from various fields. The main explanations on the motives and the extent of such transfers have been mainly sought at the individual and policy level. At the individual level, these relate mostly to the motives behind providing gifts and care whereas at the policy interest research has focused on the availability of the publicly provided formal care arrangements, (as it is often argued that the formally provided care and the size of the welfare state can discourage such transfers).

Psychologists, anthropologists, sociologists and economists have explored the individual motives and the dynamics of transfers. In fact, arguments sustaining the motivation behind informal giving to family and friends have always orbited around altruistic and non-altruistic motives [17–28] (see also Serge-Christophe Kolm [29] for a more comprehensive review of the motives behind transfers and their classifications). Altruistic motives have been more popular among non-economists in explaining prosocial behaviour (giving to others in situations when there are no immediate or visible gains). In social sciences and social psychology altruistic motives are broadly identified with acts that decrease the direct fitness of an actor while benefiting one or more recipients [30, 31]. However, in the last decades altruistic motives have become more accepted also among economists. Gary Becker, for instance, argued that there is something else beyond self-interest and this is related to genetic selection and altruism [18, 20]. The economic literature describes the altruism model as a model where for example parents care for the wellbeing of their children, or in other words they receive utility not only from their own consumption but also from consumption of their children. Consequently, the parent may choose to transfer resources to needier family members because of altruism. A distinguished feature of this model is the fact that over the lifetime a needy member of the family will receive more than she gives. If this hypothesis is empirically corroborated, then some characteristics of the needy receiver should be directly related to the extent of gifts and care (like a drop in income, a sudden illness leading to psychological or financial dependency, etc.).

Non-altruistic arguments for informal gifts and care, on the other hand, are mostly related to normative motivation (like moral obligations), social effects (as derivative of praise, esteem or gratitude [32]), social relations and social status of the giver, or simply self-interest [29]. In this later case, the costs involved with the giving are expected to be compensated (or even overcompensated) directly to the giver. There are different theoretical explanations for the motivations behind self-interested behaviour but the more predominant ones are the ‘exchange’ theory [23, 27, 33–35], the ‘strategic bequests’ motives [36–38] and ‘reciprocity’ [14, 17, 39, 40]. For instance, Cox [41] in trying to test altruism empirically using data from the US President's Commission on Pension Policy Survey sustained the ‘exchange’ theory. He suggested that utility of the giver is not only dependent on the consumption of both herself and the recipient (as the altruism model suggests), but also depends on services received by the recipient. The types of services referred to by Cox are mostly help to the donor (parents in his case) with home produced goods. The basic concept is that people pursue their self-interest through exchanging within the family and that this is enforced by explicit economic incentives. On the other hand, Bernheim et al. [42] have looked at the ‘strategic bequest’ motive focusing on bequests that parents leave to their offspring (children support their old-age parents so they can receive money/property after their death). Exchange can take the form of a ‘delayed exchanged’ (parents invest in children when they are young and they “repay” back when grown up), and the ‘direct exchange’ (children and parents exchange goods and support in the same time – e.g., services for transfers).

Another prominent non-altruistic motivation behind informal giving and receiving is also ‘reciprocity’. This is usually defined as “treating the others the same way they did treat you, just because of this particular fact and not as a result of an expected or pre-agreed exchange” [14]. Polanyi [43] has been one of the first researchers in social sciences to stress the point that reciprocity in gift giving differs from the strictly market exchange in economics. The difference with the concept of exchange (‘quid pro quo’) is that reciprocity proceeds from a set of “internal” obligations (e.g. to give, to receive, and to give back) driven by norms or collective values, and group or social pressure [14, 44]. In fact, Kolm argues that a family is neither a ‘paternalistic entity’ á la Becker and nor an ‘exchange entity’ á la Chiappori,^1^ but it represents a dense and intense network of various reciprocities in sentiments and conduct. In this context, the commands and exchanges are embedded in larger relations of reciprocity among the family members [14].

At the policy level, the expansion of the welfare state during the past 50 years has led to an increase in formal and institutionalized care and support arrangements financed by compulsory solidarity contributions in the form of tax and social insurance premiums [45]. This has resulted in a rapid increase in the cost of health care and social support systems [7]. Consequently, many countries try to shift away from solidarity arrangements based on entitlements to formal care and encourage forms of informal solidarity arrangements based on voluntary and in kind contributions. However, due to the informal and voluntary nature of informal care and private gifts, governments are much less able to ensure that sufficient care and social support is available than in the institutionalized and formalized care and support settings. The extent and generosity of the welfare system and the intervention of the state also influence the extent of informal arrangements, and hence the extent of private services and money transferred privately. A substitution effect of private transfers/help by publicly provided help would support the “crowding-out” theory [46]. Yet, many studies on intra-family transfers [47–50] have observed that even in those countries where such public transfers/services are available, private transfers do not completely disappear [51]. This raises questions on how much informal help is provided, who provides this help, who receives it, to what extent are the receivers also providers of help, and how much is this different across countries with different systems for provision of formal care.

## Methods

We use data from the fourth wave of the SHARE database [52, 53]. SHARE covers various aspects of life and health of the population aged 50 years or older. The total sample for the European countries in wave 4 included 58,489 individuals. The data were gathered in 2010 and 2011 in 16 European countries (Austria, Germany, Sweden, Netherlands, Spain, Italy, France, Denmark, Switzerland, Belgium, Czech Republic, Poland, Hungary, Portugal, Slovenia and Estonia) [52, 53]. SHARE provides information on informal help and care, financial gifts to and from members outside the household and various indicators of the social network [52, 53]. The decision to use the fourth wave of SHARE (rather than the more recent fifth wave) was based primarily on the availability of the physical proximity variables and the possibility to link this information to particular members of the network. The distance proxy is particularly important as many studies have shown that geographical distance may represent an important cost factor that could significantly influence the amount of informal care delivered, [10, 11] and therefore decreases the contact with people in the network [54]. For validity purposes, we also test our results separately using data from the fifth wave (collected in 2013 for 14 European countries - Austria, Germany, Sweden, Netherlands, Spain, Italy, France, Denmark, Switzerland, Belgium, Luxembourg, Czech Republic, Slovenia and Estonia – and Israel), using pooled data from the fourth and fifth waves. We also test our results on panel data constructed from both waves four and five of the SHARE data (all﻿ the results are presented in the Additional file 1: Tables S3 – S5).

In SHARE, the questions on informal care ask about personal care or practical household help given or received to/from any persons outside the household (from a family member, friend or neighbour) to the respondent or the partner.^2^ SHARE also asks about the financial or material gifts larger than 250 Euros given or received to/from any persons within or outside the households (including money, or transfers to cover for specific types of costs like medical care, schooling, a down payment for a home, etc.). Based on this information, the main dependent variables in our study are two: (i) a dummy variable on receiving and giving informal care from/to the persons outside the household in the last 12 months before the interview, (ii) a dummy variable on whether the respondent received or gave financial gifts of more than 250 Euros.

Receiving and giving are often correlated to each other, i.e., people who are likely to receive are also often like to give and vice versa. Yet, people who receive and give transfers may be systematically different from those who do not. If receiving and giving are estimated separately the correlation between them is not captured and this may also increase the risk of having biased estimators. To address this problem we employ a unique approach by creating a 16 cells matrix which combines the giving and receiving of both informal care and financial gifts. Hence, in terms of informal care, one could neither receive nor give it, only receive it, could only give it, or both receive and give it. The same could be said for the financial gifts. This gives us the opportunity to create a new variable capturing every possible combination, (as shown in Table 1) between the giving and receiving (or not) of informal care and gifts.

**Table 1:** The ‘matrix-like’ transformation of the giving and receiving variables

	Informal care: neither receives nor gives	Informal care: only receives	Informal care: only gives	Informal care: both receives and gives
Financial gift: neither receives nor gives	*(1) Neither care & Neither financial*	*(2) Receives care & Neither financial*	*(3) Gives care & Neither financial*	*(4) Both care & Neither financial*
Financial gift: only receives	*(5) Neither care & Receives financial*	*(6) Receives care & Receives financial*	*(7) Gives care & Receives financial*	*(8) Both care & Receives financial*
Financial gift: only gives	*(9) Neither care & Gives financial*	*(10) Receives care & Gives financial*	*(11) Gives care & Gives financial*	*(12) Both care & Gives financial*
Financial gift: both receives and gives	*(13) Neither care & Both financial*	*(14) Receives care & Both financial*	*(15) Gives care & Both financial*	*(16) Both care & both financial*

After this transformation our dependent variable could fall within any of the categories in Table 1 representing any of the combinations of transfers. For instance, category 1 ‘neither care – neither financial’ corresponds to individuals who declared to ‘neither receiving nor giving informal care’ and ‘neither receiving nor giving financial gifts’. Our outcome of interest *Y* could take any *n* values such as *n = 1,2,3,…16.*

In terms of hypothesis testing, we were interested to check for evidence supporting either ‘altruistic’ or the ‘non-altruistic’ theories. For instance, if receiving a type of transfer is not associated with the giving of another type (e.g. ‘receives care – neither financial’; ‘gives careneither financial’; ‘neither care – receives financial’; ‘neither care- gives financial’) this supports a more altruistic motive behind the transfers. On the other hand, the ‘exchange’ theories are supported more if receiving is conditional on giving (such as in ‘receives care – gives financial’ or ‘gives care - receives financial’). Instead, the ‘reciprocity’ theory is supported more if we observe giving and receiving of the same type (i.e., ‘both care – neither financial’ or ‘neither care – both financial’).

We were also interested in checking the main characteristics that are associated with giving and receiving. For this we assume that ${X}_i$ is a vector of exogenous variables, like age, gender, household size, having the partner in the same household, instrumental activities of daily living (IADL) scales, having been hospitalized in the last 12 months, income quintile, employment status, the share of people in the network living less than 5 km squared away, and country of residence. The choice of these variables was based on the availability of them in the SHARE data as well as on previous studies which have shown that they all affect the probability of informal care or gifts to other family members or friends [2, 9, 11, 47, 49, 55–58]. Furthermore we assume ${\beta}^{(i)}$ is a set of vectors of parameters that correspond to each of the outcomes specified in Table 1 (for each *i = 1,2,3. …,16*). Our focus is on checking how the relationship between giving and receiving differs by age, gender and distance to the relatives and friends. To control for all these factors we estimated a multinomial logit regression [59, 60] such that the probability $\left({\pi}_i\right)$ for each of the outcomes $\left(i=1,2, \dots, 16\right)$ is determined as:

1 $$ \begin{array}{l}{\pi}_1\left(y=1\right)=\frac{e^{X{\beta}^{(1)}}}{{\boldsymbol{e}}^{\boldsymbol{X}{\boldsymbol{\beta}}^{(1)}}+{e}^{X{\beta}^{(2)}}+\dots +{e}^{X{\beta}^{(16)}}}\hfill \\ {}{\pi}_2\left(y=2\right)=\frac{e^{X{\beta}^{(2)}}}{e^{X{\beta}^{(1)}}+{e}^{X{\beta}^{(2)}}+\dots +{e}^{X{\beta}^{(16)}}}\hfill \\ {}\dots \dots \hfill \\ {}{\pi}_{16}\left(y=16\right)=\frac{e^{X{\beta}^{(16)}}}{e^{X{\beta}^{(1)}}+{e}^{X{\beta}^{(2)}}+\dots +{e}^{X{\beta}^{(16)}}}\hfill \end{array} $$

For comparative purposes, we set the outcome number 1, ‘neither care – neither financial’, as the reference category. Hence, if we set ${\beta}^{(1)}=0$ the other set of coefficients simply measure the change relative to $y=1$, and the probabilities will be estimated as:

2 $$ \begin{array}{l}{\pi}_1\left(y=1\right)=\frac{1}{1+{e}^{X{\beta}^{(2)}}+\dots +{e}^{X{\beta}^{(16)}}}\hfill \\ {}{\pi}_2\left(y=2\right)=\frac{e^{X{\beta}^{(2)}}}{1+{e}^{X{\beta}^{(2)}}+\dots +{e}^{X{\beta}^{(16)}}}\hfill \\ {}\dots \dots \hfill \\ {}{\pi}_{16}\left(y=16\right)=\frac{e^{X{\beta}^{(16)}}}{1+{e}^{X{\beta}^{(2)}}+\dots +{e}^{X{\beta}^{(16)}}}\hfill \end{array} $$

And the relative probability of outcome *i* to the reference outcome 1 (neither-neither):

3 $$ \frac{\pi_i\left(y=i\right)}{\pi_1\left(y=1\right)}={e}^{X{\beta}^{(i)}}\kern2em \left(i= 1, 2, 3. \dots, 1 6\right) $$

One of the limitations of the SHARE dataset is that it only indicates that a relative or friend (e.g. as a partner, child, mother or father, other relative, friend, etc.) was involved but it does not identify the specific person who gave or received money or help. Therefore we cannot link the information on specific transfers (giving and receiving of informal care or financial gifts) to a specific person. This may limit the interpretation of our results on the altruistic or non-altruistic theories as: (i) we are not sure if respondents are referring to the same person ﻿﻿﻿to/from whom they gave and received﻿﻿﻿, and (ii) we cannot control for characteristics of persons who are providing (or receiving) care or gifts. To overcome the first problem we check the validity of the results by repeating the same analysis for distinct groups of relatives, namely: the children, close relatives, other relatives and friends (results for children are given in the Additional file 1: Table S2 and all other results are available from the authors).

## Results

### Descriptive statistics

We have data on a total number of 35,249 individuals who reported on both receiving and giving of informal care and financial gifts in the last 12 months. Table 1 shows that giving financial gifts (of 250 Euros or more) and giving informal practical help or personal care to persons outside the household had the highest overall mean incidence when the data for all countries are pooled together: 24.4 % of all respondents have given financial gifts and 19.0 % have provided help or care in the last 12 months. The table also shows that a relatively small share of the respondents both gives and receives informal care (5.6 %) or both gives and receives financial gifts (3.1 %).

A relatively large share of respondents (46.5 %) belongs to the ‘*neither care – neither financial*’ group as they neither give nor receive informal care and neither give nor receive financial gifts. On the other hand, there is a much lower share of respondents at the other extreme ‘*both care – both financial*’ as only 0.5 % of them give and receive both informal care and financial gifts. Another interesting trend is also the share of respondents that fall under *‘gives care – neither financial’* which is about 10.6 % of sample. Similarly, people who fall under *‘neither care – gives financial’* accounted for 13.5 % of the total sample.

A more detailed overview for each of the countries is given in Additional file 1: Table S1. Our data show that there are no clear patterns between countries and there is a relatively large heterogeneity in terms of patterns of receiving and giving informal care and financial gifts between countries in Europe. In general, Northern and Western European countries have higher incidences of giving care or financial gifts while Southern and Eastern European (SEE) countries in receiving them. Hence, countries like Denmark, Sweden and Netherlands have higher incidences than the mean in*’giving care – neither financial*’ while Austria, Germany, Sweden and Switzerland have higher incidences for ‘*neither care – giving financial*’. Denmark, Germany and Sweden also have higher incidences for ‘*receiving care – giving financial*’. SEE countries have instead the highest incidences for ‘*receiving care – neither financial*’ (Spain, Czech Republic, Estonia, Hungary) as well as ‘*neither care – receiving financial*’ (Italy, Estonia, Poland).

### Determinants of receiving and giving informal help and care

Table 2 shows the results of the multinomial model for selected variables including age dummies, gender and share of social network members living within 5 km squares (a complete table of results is provided in Additional file 1: Table S3).

**Table 2:** Descriptive Statistics: the incidence of giving and receiving care and financial help

	Informal care: neither receives nor gives	Informal care: only receives	Informal care: only gives	Informal care: both receives and gives	Total
Financial gift: neither receives nor gives	0,465	0,087	0,106	0,030	0,688
	(0,007)	(0,004)	(0,004)	(0,003)	(0,006)
Financial gift: only receives	0,017	0,007	0,009	0,004	0,037
	(0,002)	(0,001)	(0,001)	(0,001)	(0,003)
Financial gift: only gives	0,135	0,027	0,065	0,016	0,244
	(0,005)	(0,003)	(0,003)	(0,002)	(0,006)
Financial gift: both receives and gives	0,014	0,002	0,010	0,005	0,031
	(0,001)	(0,000)	(0,001)	(0,001)	(0,002)
*Total*	0,631	0,123	0,190	0,056	
	(0,007)	(0,005)	(0,005)	(0,004)	

Standard deviations in brackets

The results show that older age cohorts (between 65 and 75 or higher than 75 years old) are more likely to receive help from outside the household (see Table 3A and B) compared to those younger than 65 years old. At the same time these older cohorts are less likely to give help outside of the household or receive and give financial help. An interesting trend is that the coefficient for the category ‘*receives care – neither financial’* increases with age indicating that informal care received increases with age even if no financial transfers are given in return.

**Table 3:** Selected coefficients of the multinomial regression model for selected variables

	Informal care			
Fin. gifts	*Neither receives nor gives*	*Only receives*	*Only gives*	*Both receives and gives*
A Age between 65 and 75 years old (ref: younger than 65)				
*Neither receives nor gives*	-	0.166^b^	−0.363^c^	−0.220^c^
		(0.068)	(0.050)	(0.085)
*Only receives*	−0.146	0.052	−0.479^c^	−0.373
	(0.122)	(0.202)	(0.174)	(0.252)
*Only gives*	−0.024	0.262^b^	−0.461^c^	−0.372^c^
	(0.049)	(0.117)	(0.066)	(0.121)
*Both receives and gives*	−0.049	0.406	−0.637^c^	−0.674^c^
	(0.130)	(0.248)	(0.156)	(0.186)
B Age older than 75 years old (ref: younger than 65)				
*Neither receives nor gives*	-	0.934^c^	−1.012^c^	−0.546^c^
		(0.066)	(0.067)	(0.100)
*Only receives*	−0.508^c^	0.106	−1.129^c^	−0.787^c^
	(0.149)	(0.208)	(0.240)	(0.301)
*Only gives*	−0.083	0.804^c^	−1.220^c^	−0.469^c^
	(0.058)	(0.117)	(0.099)	(0.141)
*Both receives and gives*	−0.228	0.397	−1.328^c^	−1.128^c^
	(0.166)	(0.274)	(0.248)	(0.251)
C Gender: female				
*Neither receives nor gives*	-	0.312^c^	0.235^c^	0.253^c^
		(0.049)	(0.039)	(0.067)
*Only receives*	0.431^c^	0.857^c^	0.613^c^	0.874^c^
	(0.097)	(0.183)	(0.130)	(0.206)
*Only gives*	−0.098^c^	0.090	0.164^c^	0.183^b^
	(0.037)	(0.082)	(0.049)	(0.085)
*Both receives and gives*	0.237^b^	0.667^c^	0.528^c^	0.301^b^
	(0.096)	(0.203)	(0.109)	(0.139)
D Share of people in the social network living less than 5 km away				
*Neither receives nor gives*	-	0.402^c^	0.497^c^	0.621^c^
		(0.057)	(0.053)	(0.084)
*Only receives*	−0.050	0.419^b^	0.200	0.222
	(0.125)	(0.178)	(0.169)	(0.227)
*Only gives*	0.127^b^	0.593^c^	0.482^c^	0.538^c^
	(0.053)	(0.102)	(0.071)	(0.117)
*Both receives and gives*	0.231^a^	−0.029	0.498^c^	0.615^c^
	(0.139)	(0.239)	(0.152)	(0.182)
Pseudo R-squared (0.114)				
Number of observations (35,363)				

Other control variable included: ‘household size’, ‘having the partner in the same household’, ‘self-perceived health score, ‘activities of daily living (ADL) scale’, ‘having been hospitalized in the last 12 months’, ‘income quintiles’, ‘employment and retirement status’, and a list of dummies for the country of residence.; Standard deviations in brackets; ^a^significant at 10 %; ^b^significant at 5 %; ^c^significant at 1 %

However, so does the coefficient for the category ‘*receives care – gives financial’*, which especially for individuals older than 75 years increases quite substantially and becomes statistically significant at the 99 % confidence level. The results show that for both age categories giving of financial gifts is associated with receiving informal help (‘*receives care – gives financial*’). For the other categories coefficients are either negative or not statistically significant.

‘Reciprocity’ of informal care (‘*both care – neither financial*’) appears to decrease with age, probably due to the fact that the old age cannot provide much informal care in return.

Results for gender show that women are more likely than men to receive and give practical help and care outside the household, as well as give and receive financial help (though the results for giving financial gifts were not statistically significant). The result for the coefficients of category ‘*receives care – neither financial*’ is positive and statistically significant and so is the coefficient for ‘receives care – receives financial’ showing that women are more likely than men to receive either just care or both care and financial help. Interestingly, the coefficient for ‘*receives care – gives financial*’ is not statistically significant whereas ‘*gives care – receives financial*’ is positive and statistically significant showing that women may be more likely than men to give care and receive financial help rather than the other way around. This may be related to the fact that women may have less financial means to provide financial help to others.

However, despite the lower probability for financial transfers, women appear to be more ‘reciprocal’ than men in both informal care and financial gifts. Both coefficients for categories ‘*both care – neither financial*’ and ‘*neither care – both financial*’ are positive and statistically significant.

Having a larger share of network members in a distance closer than 5 km squares increases the probability of both receiving and giving informal care and also giving financial gifts but does not always have a statistically significant effect on receiving financial help. Again, it is interesting to note that having a larger share of social network members closer than 5 km increases both the probability of ‘*receives care – neither financial*’ and ‘*receives care – gives financial’*. This shows that people with a higher share of their network living nearby are more likely to receive informal care either “in return” for financial gifts or not. Closer proximity of the network members also increases ‘reciprocal’ informal care as the coefficient for the category ‘*both care – neither financial*’ is positive and statistically significant at 99 % confidence level.

The effect of other control variables is as expected (see Additional file 1: Table S3). Hence, having a partner in the household generally reduces the probability of receiving and giving both care from outside and financial help. Similarly, a larger household size reduces the probability of getting help from outside the household and increases the probability of giving/receiving help inside the household. Having been in the hospital during the last 12 months and having limitations with IADL has more or less the same effect on help and care (increasing the probability of receiving help from outside and inside the household and reducing the probability of giving help outside the household – but yet not inside the household). In general, being retired, unemployed, receiving disability benefits or being a homemaker decreases the probability of giving financial help compared to being employed. This is to be expected as these groups have in general less disposable income than the employed. This result is also supported by the results on the particular income quintiles (the lower four quintiles were less likely to have given financial help compared to the highest one).

The results for the country dummies show that there does not exist a clear pattern between countries. Responders in some Central European and SEE countries (like Switzerland, France, Portugal, Spain, Hungary, Slovenia) have a higher probability of receiving informal care help both when giving and not giving financial transfers (i.e., the ‘*receives care – neither financial*’ and ‘*receives care – gives financial*’ categories) if compared to others, but as mentioned, the patterns are not consistent over the countries.

The validity checks on the particular transfers from and to children (see Additional file 1: Table S2) mainly confirm the results from the total sample with the only difference that here the categories ‘*receives care – receives financial’* (especially for people older than 75 years old) becomes positive and statistically significant at 99 % confidence level. Results show that the relationship with children is a usually oriented towards receiving care either together with receiving and giving financial gifts or not (i.e., coefficients for categories ‘*receives care - neither financial’; ‘receives care - receives financial’; ‘receives care - gives financial’* are positive and statistically significant) but less in giving care.

In order to further check the robustness of our results we have also run our models on the cross-sectional data from the fifth wave of SHARE, on a pooled sample from waves four and five and on the (unbalanced) panel data from waves four and five. It should be noted that some of such models were run without one (i.e. the variable for geographical proximity of the network members) or more of the original independent variables. All results were consistent with our findings (see Additional file 1: Tables S3 – S5) and confirm the robustness of the relations presented here.

## Discussion

Receiving and giving informal care or financial help are not necessarily separate acts. They depend on the context, which includes the motivation behind the act of helping and the availability of alternative forms of help, such as formal care, insurance coverage and social transfers. Personal motives are usually divided in altruistic reasons (the willingness to give informal care or financial transfers when observing a need or any other valid reason for helping without expecting anything in return) and non-altruistic reasons (when giving is based on the expectation that the costs involved will be compensated to the giver at some point in time). Previous research has also highlighted that the extent of solidarity in a society may also be linked to the formal provision of long-term care since state intervention may (partially) contribute to a ‘crowding-out’ of private transfers [46]. Yet, informal and formal care are not perfect substitutes [11, 12] and moreover, people who receive care are also important support providers [15, 61]. This paper has explored the complex nature of informal care and financial help transferred by and to older people taking into account the reciprocal nature of informal transfers as well as the differences that may exist across the 16 European countries in the SHARE database.

The data used in this study came from a cohort of people aged 50 and plus [52, 53]. Despite the older age of the participants in the sample, our results show that the informal giving of personal care to persons outside the household or financial gifts is the most prevalent transfer. This confirms the hypothesis that this population group remains an important support provider [15] in all the countries in the survey. In fact, previous studies have found that monetary transfers within a family flow primarily from older to younger generations [2, 23, 25, 27, 47, 62, 63]. Albertini et al. [47] show that, though the probability of giving decreases at very old age, even the individuals above 70 years old remain net givers.

Our findings suggest that the elderly (especially categories over 75 years old) are also important receivers of informal care and this is “divided” between those who give financial and receive care (‘*receives care – gives financial’*) and those who receive care without giving any financial gifts (‘*receives care – neither financial’)*. While the later finding would support a more ‘altruistic’ motive behind transfers, the earlier finding suggests an ‘exchange’ motive. Previous studies have documented that elderly parents are more likely to give financial gifts to children providing informal care [13, 23, 33, 58] but that also there may be things beyond the pure ‘exchange’ theory [27]. In fact, the conclusion about the ‘altruistic’ motives should be interpreted more cautiously as relations among the members of social networks tend to be more dynamic and also change over time (e.g. relatives may have provided care or gifts to each-other in the past or may do so in the future). Moreover, the absence of formal care arrangements may elicit caregiving activities by others. The lack of adequate formal arrangements has been argued to be a driving factor for informal caregiving especially in Eastern [64] and Southern European countries [47]. On the other hand, the widespread availability and extent of formal care and support programs may crowd out the need for informal care. These two factors may explain regional differences in informal caregiving [65]. Several of the countries in this study have formal support programs in place where, for instance, children or volunteers are compensated for their time spent in caregiving activities. However, the set-up, conditions and benefits of the support programs differ between countries [2, 55, 65]. It is argued that the existence of such interventions together with the socio-economic differences may also change social norms and the way people help each-other [65].

One other important determinant of informal care we examine here is gender. We find that elderly women are less likely to experience ‘exchange-like’ transfers (‘*receives care – gives financial’*) and more likely to receive ‘altruistic-like’ transfers (‘*receives care – neither financial’* and ‘*receives care – receives financial’)*. Previous studies have indicated that women are more likely to behave altruistically than men [66]. However, this result may also be related to the fact that women are more disadvantaged financially than men and therefore have less financial means. Our results show that women appear to be important providers of informal care as they are more likely than mean to provide care either in the presence of gifts ‘*gives care – receives financial’* or not ‘*gives care – neither financial’*.

Women are also more likely to be ‘reciprocal’ than men in both informal care and financial gifts as they are more likely to simultaneously receive and give informal care without receiving or giving financial (and the other way around). Previous studies also have shown that there may be gender differences when it comes to reciprocity [67].

An important factor in giving and receiving informal care is also physical proximity with the members of the social network. In fact, we find here that distance matters for both receiving and giving informal care and for the ‘reciprocity’ of such transfers.

In terms of cross country differences, our results show that the incidence of informal care remains high even in those countries where long-term and old-age care services are almost entirely based on public provision of formal care and support (like in the Scandinavian countries and the Netherlands). In fact, data from 2012 on long-term care expenditure show that countries like the Netherlands, Sweden and Denmark are among the countries with the highest share of expenditure on long-term care as a percentage of GDP (with Netherlands spending a total of about 4.1 % and Sweden and Denmark 3.7 and 2.6 respectively) [68]. The Czech Republic, Estonia and Portugal on the other hand are amongst the countries spending the lowest share of GDP (0.2 to 0.3 % of the GDP) with expenditures in the other countries somewhere in between [68]. The numbers on the incidence of informal care to some extent go against the theory of the ‘crowding-out’ of private transfers. In fact, previous studies [11] have shown that, for instance transfers between children and parents are less frequent in Southern European countries compared to the Nordic countries (Sweden and Denmark) but they can be more intense in nature [47]. The same studies show that the Continental European countries are somewhere in between the two, which is also confirmed by our results.

Yet, we could not identify any distinguished patterns between countries when it comes to the association of informal care and financial gifts. Previous studies have also found similarities in terms of transfers patterns of money and care between the European countries [15, 47]. This confirms that despite the differences there may be some common patterns when it comes to who receives more and how are the transfers of informal care and financial gifts interrelated with one-another.

## Conclusions

The results of the analysis in this paper show that despite the differences between the selected European countries, the overall incidence of transfers of informal care and financial gifts remain high among the 50 plus years old. Moreover we have shown that there are a few clear patterns among European countries in terms of the interrelation of giving and receiving informal help and financial gifts. The general positive relationship between giving informal care and receiving financial gifts suggest some form of ‘exchange’ between the elderly and their relatives. On the other hand, the positive relationship between receiving informal help without transferring financial gifts suggest other motivations that go beyond the exchange theory. We also found that the combination(s) of giving and receiving is more enhanced with age, gender and physical proximity of the relatives and friends. However, ‘reciprocal-like’ of the same transfers are more likely for women and relatives with a closer ﻿distance network.

To our knowledge, this paper is the first to study the different combinations of receiving and giving of informal care and financial gifts using nationally representative data for people 50 year plus in multiple European countries. From a policy perspective, our results show that the motives behind giving and receiving may not be influenced much by the different arrangements of formal care available in the countries included in our analysis. Yet, more attention should be paid to how such relations change depending on the type of care delivered and the intensity of informal care. Our results call also for more attention on analysing the effect of informal help especially among certain groups like older generations, the worse off, as well as people having a more physically distant social network. Further research should be dedicated to exploring the situations leading to the ‘altruistic-like’ and ‘exchange-like’ combination of transfers. This is especially important as the ‘exchange-like’ transfers may be much more prone to the ‘crowding-out’ effect on both ends, i.e., both from the giver and the receiver perspectives.

## Additional files

Additional file 1:
**Table S1.** Descriptive Statistics: The incidence of giving and receiving care and financial help by country. **Table S2.** Selected coefficients of the multinomial regression model for selected variables on transfers from and to children. **Table S3.** Selected coefficients of the multinomial regression model for selected variables – Wave 5 of SHARE data. **Table S4.** Selected coefficients of the multinomial regression model for the pooled data. **Table S5.** Coefficients of the multinomial regression model for the multinomial logistic model with random effects– Wave 4 and 5 of SHARE data. **Table S6.** Full results for the multinomial regression model for transfers from and to children. (DOCX 73 kb)

## Abbreviations

IADL: Instrumental activities of daily living
SEE: Southern and Eastern European
SHARE: Survey of health, ageing and retirement in Europe

## Footnotes

1 See also Chiappori [21] and Browning et al. [69].

2 SHARE also provides information on the regular (daily or almost daily during at least three months) personal care (washing, getting out of bed, or dressing) given or received to/from any persons living in the same household. However, this is mostly provided between the households members and therefore it was not of interest for this paper.
