# Supplementary material for: Informal care and gifts to and from older people in Europe: The interlinks between giving and receiving
Source: BMC Health Serv Res. 2016 Oct 21;16:603. doi: 10.1186/s12913-016-1830-7 (PMC5073444; doi:10.1186/s12913-016-1830-7)
Supplement: Additional file 1: — Table S1. Descriptive Statistics: The incidence of giving and receiving care and financial help by country. Table S2. Selected coefficients of the multinomial regression model for selected variables on transfers from and to children. Table S3. Selected coefficients of the multinomial regression model for selected variables – Wave 5 of SHARE data. Table S4. Selected coefficients of the multinomial regression model for the pooled data. Table S5. Coefficients of the multinomial regression model for the multinomial logistic model with random effects– Wave 4 and 5 of SHARE data. Table S6. Full results for the multinomial regression model for transfers from and to children. (DOCX 73 kb) [file 12913_2016_1830_MOESM1_ESM.docx]

**Table S1.** Descriptive Statistics: The incidence of giving and receiving care and financial help by country

|  | *(1) Neither care & Neither financial* | *(2) Receives care & Neither financial* | *(3) Gives care & Neither financial* | *(4) Both care & Neither financial* | *(5) Neither care & Receives financial* | *(6) Receives care & Receives financial* | *(7) Gives care & Receives financial* | *(8) Both care & Receives financial* | *(9) Neither care & Gives financial* | *(10) Receives care & Gives financial* | *(11) Gives care & Gives financial* | *(12) Both care & Gives financial* | *(13) Neither care & Both financial* | *(14) Receives care & Both financial* | *(15) Gives care & Both financial* | *(16) Both care & both financial* |
| --- | --- | --- | --- | --- | --- | --- | --- | --- | --- | --- | --- | --- | --- | --- | --- | --- |
| *Austria* | *0.458* | *0.079* | *0.082**** | *0.023**** | *0.019* | *0.005* | *0.011* | *0.003* | *0.162**** | *0.025* | *0.658**** | *0.102**** | *0.078**** | *0.015**** | *0.014**** | *0.002*** |
|  | *[0.498]* | *[0.270]* | *[0.275]* | *[0.149]* | *[0.138]* | *[0.073]* | *[0.103]* | *[0.058]* | *[0.368]* | *[0.155]* | *[0.474]* | *[0.303]* | *[0.268]* | *[0.121]* | *[0.117]* | *[0.050]* |
| *Belgium* | *0.386**** | *0.081* | *0.186**** | *0.042**** | *0.014* | *0.003*** | *0.012*** | *0.004* | *0.110**** | *0.025* | *0.089**** | *0.019* | *0.010*** | *0.003* | *0.012* | *0.004*** |
|  | *[0.487]* | *[0.273]* | *[0.389]* | *[0.200]* | *[0.117]* | *[0.059]* | *[0.110]* | *[0.066]* | *[0.313]* | *[0.157]* | *[0.285]* | *[0.135]* | *[0.101]* | *[0.050]* | *[0.110]* | *[0.061]* |
| *Denmark* | *0.270**** | *0.080* | *0.150**** | *0.088**** | *0.006**** | *0.005* | *0.012* | *0.008*** | *0.128* | *0.033**** | *0.100**** | *0.063**** | *0.013* | *0.004* | *0.020**** | *0.019**** |
|  | *[0.444]* | *[0.272]* | *[0.358]* | *[0.284]* | *[0.078]* | *[0.073]* | *[0.109]* | *[0.091]* | *[0.334]* | *[0.179]* | *[0.300]* | *[0.243]* | *[0.113]* | *[0.061]* | *[0.141]* | *[0.136]* |
| *Germany* | *0.392**** | *0.076* | *0.110* | *0.034* | *0.012* | *0.006* | *0.011* | *0.004* | *0.171**** | *0.042**** | *0.077** | *0.028*** | *0.017* | *0.001* | *0.012* | *0.007* |
|  | *[0.488]* | *[0.265]* | *[0.313]* | *[0.181]* | *[0.107]* | *[0.079]* | *[0.102]* | *[0.065]* | *[0.377]* | *[0.201]* | *[0.267]* | *[0.164]* | *[0.129]* | *[0.033]* | *[0.107]* | *[0.086]* |
| *Netherlands* | *0.440*** | *0.055**** | *0.144**** | *0.042** | *0.012* | *0.003* | *0.010* | *0.007** | *0.126* | *0.025* | *0.093**** | *0.025** | *0.004**** | *0.002* | *0.011* | *0.001**** |
|  | *[0.497]* | *[0.229]* | *[0.351]* | *[0.200]* | *[0.109]* | *[0.056]* | *[0.097]* | *[0.083]* | *[0.332]* | *[0.155]* | *[0.290]* | *[0.155]* | *[0.067]* | *[0.044]* | *[0.106]* | *[0.025]* |
| *Sweden* | *0.339**** | *0.072** | *0.148**** | *0.042* | *0.011* | *0.002*** | *0.011* | *0.002* | *0.158**** | *0.032*** | *0.130**** | *0.026*** | *0.009* | *0.004* | *0.007* | *0.008* |
|  | *[0.473]* | *[0.259]* | *[0.355]* | *[0.200]* | *[0.103]* | *[0.040]* | *[0.103]* | *[0.050]* | *[0.364]* | *[0.176]* | *[0.336]* | *[0.160]* | *[0.095]* | *[0.064]* | *[0.086]* | *[0.090]* |
| *Switzerland* | *0.486*** | *0.050**** | *0.122* | *0.019**** | *0.013* | *0.003*** | *0.010* | *0.001*** | *0.144**** | *0.016*** | *0.089**** | *0.014* | *0.017* | *0.002* | *0.011* | *0.003**** |
|  | *[0.500]* | *[0.218]* | *[0.328]* | *[0.138]* | *[0.112]* | *[0.050]* | *[0.098]* | *[0.036]* | *[0.351]* | *[0.126]* | *[0.284]* | *[0.119]* | *[0.129]* | *[0.046]* | *[0.106]* | *[0.050]* |
| *France* | *0.493**** | *0.065**** | *0.142**** | *0.020**** | *0.012*** | *0.002**** | *0.009* | *0.001**** | *0.148**** | *0.013**** | *0.067* | *0.012**** | *0.007**** | *0.001**** | *0.006**** | *0.001**** |
|  | *[0.500]* | *[0.247]* | *[0.350]* | *[0.140]* | *[0.108]* | *[0.045]* | *[0.094]* | *[0.030]* | *[0.355]* | *[0.115]* | *[0.250]* | *[0.107]* | *[0.080]* | *[0.034]* | *[0.077]* | *[0.038]* |
| *Italy* | *0.452* | *0.077* | *0.094**** | *0.019**** | *0.021** | *0.012**** | *0.006* | *0.002* | *0.166**** | *0.026* | *0.077**** | *0.007**** | *0.024**** | *0.001** | *0.011* | *0.003*** |
|  | *[0.498]* | *[0.267]* | *[0.291]* | *[0.137]* | *[0.144]* | *[0.110]* | *[0.080]* | *[0.049]* | *[0.372]* | *[0.161]* | *[0.266]* | *[0.085]* | *[0.152]* | *[0.038]* | *[0.106]* | *[0.054]* |
| *Portugal* | *0.609**** | *0.080* | *0.090**** | *0.021*** | *0.015* | *0.003* | *0.004** | *0.003* | *0.094**** | *0.015** | *0.039**** | *0.007**** | *0.009** | *0.004* | *0.006*** | *0.001**** |
|  | *[0.488]* | *[0.272]* | *[0.286]* | *[0.144]* | *[0.120]* | *[0.057]* | *[0.064]* | *[0.057]* | *[0.292]* | *[0.120]* | *[0.194]* | *[0.081]* | *[0.094]* | *[0.064]* | *[0.075]* | *[0.029]* |
| *Spain* | *0.658**** | *0.102**** | *0.078**** | *0.015**** | *0.014**** | *0.002*** | *0.005** | *0.001*** | *0.082**** | *0.007**** | *0.022**** | *0.003**** | *0.005**** | *0.000**** | *0.002**** | *0.001**** |
|  | *[0.474]* | *[0.303]* | *[0.268]* | *[0.121]* | *[0.117]* | *[0.050]* | *[0.073]* | *[0.031]* | *[0.274]* | *[0.083]* | *[0.147]* | *[0.059]* | *[0.073]* | *[0.022]* | *[0.050]* | *[0.038]* |
| *Czech Rep.* | *0.371**** | *0.116**** | *0.090**** | *0.067**** | *0.019* | *0.019**** | *0.009* | *0.015**** | *0.099**** | *0.028**** | *0.050**** | *0.034**** | *0.023**** | *0.015**** | *0.017**** | *0.029**** |
|  | *[0.483]* | *[0.320]* | *[0.286]* | *[0.250]* | *[0.138]* | *[0.136]* | *[0.095]* | *[0.120]* | *[0.299]* | *[0.166]* | *[0.218]* | *[0.182]* | *[0.150]* | *[0.121]* | *[0.128]* | *[0.167]* |
| *Estonia* | *0.456* | *0.127**** | *0.105** | *0.039*** | *0.023**** | *0.007* | *0.009* | *0.003** | *0.104**** | *0.027*** | *0.043**** | *0.018* | *0.014* | *0.004* | *0.015** | *0.006* |
|  | *[0.498]* | *[0.333]* | *[0.307]* | *[0.194]* | *[0.149]* | *[0.082]* | *[0.094]* | *[0.051]* | *[0.305]* | *[0.161]* | *[0.204]* | *[0.134]* | *[0.119]* | *[0.066]* | *[0.120]* | *[0.079]* |
| *Hungary* | *0.538**** | *0.099*** | *0.108* | *0.036* | *0.017* | *0.008* | *0.006* | *0.003* | *0.095**** | *0.017* | *0.038**** | *0.016* | *0.010** | *0.001*** | *0.007*** | *0.002*** |
|  | *[0.499]* | *[0.298]* | *[0.310]* | *[0.185]* | *[0.131]* | *[0.089]* | *[0.079]* | *[0.058]* | *[0.294]* | *[0.129]* | *[0.191]* | *[0.125]* | *[0.097]* | *[0.034]* | *[0.082]* | *[0.047]* |
| *Poland* | *0.534**** | *0.087* | *0.073**** | *0.013**** | *0.029**** | *0.009* | *0.013* | *0.005* | *0.128* | *0.021* | *0.050* | *0.008**** | *0.015* | *0.002* | *0.012* | *0.002*** |
|  | *[0.499]* | *[0.283]* | *[0.260]* | *[0.112]* | *[0.168]* | *[0.093]* | *[0.112]* | *[0.070]* | *[0.334]* | *[0.145]* | *[0.219]* | *[0.088]* | *[0.120]* | *[0.044]* | *[0.107]* | *[0.044]* |
| *Slovenia* | *0.623**** | *0.061**** | *0.084**** | *0.011**** | *0.013* | *0.001**** | *0.005*** | *0.001*** | *0.115* | *0.013**** | *0.041**** | *0.009**** | *0.011* | *0.001*** | *0.007** | *0.002**** |
|  | *[0.485]* | *[0.240]* | *[0.277]* | *[0.106]* | *[0.115]* | *[0.032]* | *[0.068]* | *[0.032]* | *[0.319]* | *[0.115]* | *[0.198]* | *[0.096]* | *[0.106]* | *[0.032]* | *[0.085]* | *[0.045]* |
| *Total* | *0.465* | *0.086* | *0.113* | *0.034* | *0.016* | *0.006* | *0.009* | *0.004* | *0.125* | *0.022* | *0.063* | *0.019* | *0.015* | *0.004* | *0.012* | *0.007* |
|  | *[0.499]* | *[0.280]* | *[0.317]* | *[0.181]* | *[0.127]* | *[0.079]* | *[0.094]* | *[0.065]* | *[0.331]* | *[0.148]* | *[0.242]* | *[0.135]* | *[0.120]* | *[0.063]* | *[0.108]* | *[0.084]* |

*Standard deviations in brackets; * significant at 10%; ** significant at 5%; *** significant at 1%*

**Table S2.** Selected coefficients of the multinomial regression model for selected variables on transfers from and to children

| A2.A Age between 65 and 75 years old (ref: younger than 65) | A2.B Age older than 75 years old (ref: younger than 65) |
| --- | --- |
| \| **Informal**  **care**  **Fin. gifts** \| *Neither receives nor gives* \| *Only receives* \| *Only gives* \| *Both receives and gives* \| \| --- \| --- \| --- \| --- \| --- \| \| *Neither receives nor gives* \| - \| 0.553*** \| 0.125 \| 0.079 \| \|  \| (0.079) \| (0.086) \| (0.147) \| \| *Only receives* \| 0.332** \| 0.428* \| 0.431 \| 0.248 \| \| (0.132) \| (0.231) \| (0.404) \| (0.452) \| \| *Only gives* \| -0.040 \| 0.477*** \| -0.221** \| -0.211 \| \| (0.044) \| (0.138) \| (0.108) \| (0.208) \| \| *Both receives and gives* \| 0.243* \| 0.838*** \| 0.257 \| -0.415 \| \| (0.144) \| (0.284) \| (0.347) \| (0.317) \| | \| **Informal**  **care**  **Fin. gifts** \| *Neither receives nor gives* \| *Only receives* \| *Only gives* \| *Both receives and gives* \| \| --- \| --- \| --- \| --- \| --- \| \| *Neither receives nor gives* \| - \| 1.424*** \| -0.402*** \| 0.015 \| \|  \| (0.077) \| (0.112) \| (0.167) \| \| *Only receives* \| 0.166 \| 0.732*** \| -0.116 \| 0.407 \| \| (0.153) \| (0.235) \| (0.537) \| (0.493) \| \| *Only gives* \| -0.127** \| 1.099*** \| -1.154*** \| 0.101 \| \| (0.053) \| (0.139) \| (0.174) \| (0.228) \| \| *Both receives and gives* \| 0.245 \| 0.933*** \| 0.065 \| -0.160 \| \| (0.180) \| (0.316) \| (0.449) \| (0.365) \| |
| A2.C Gender: female | A2.D Share of people in the social network living more than 5 km away |
| \| **Informal**  **care**  **Fin. gifts** \| *Neither receives nor gives* \| *Only receives* \| *Only gives* \| *Both receives and gives* \| \| --- \| --- \| --- \| --- \| --- \| \| *Neither receives nor gives* \| - \| 0.658*** \| 0.365*** \| 0.717*** \| \|  \| (0.059) \| (0.071) \| (0.132) \| \| *Only receives* \| 0.853*** \| 0.968*** \| 1.386*** \| 1.346*** \| \| (0.121) \| (0.217) \| (0.420) \| (0.494) \| \| *Only gives* \| 0.067** \| 0.318*** \| 0.089 \| 0.392*** \| \| (0.032) \| (0.098) \| (0.083) \| (0.149) \| \| *Both receives and gives* \| 0.472*** \| 0.534** \| 0.850*** \| 0.251 \| \| (0.119) \| (0.231) \| (0.308) \| (0.264) \| | \| **Informal**  **care**  **Fin. gifts** \| Neither receives nor gives \| Only receives \| Only gives \| Both receives and gives \| \| --- \| --- \| --- \| --- \| --- \| \| *Neither receives nor gives* \| - \| 0.420*** \| 0.510*** \| 0.431*** \| \|  \| (0.063) \| (0.092) \| (0.148) \| \| *Only receives* \| -0.284** \| 0.190 \| 0.184 \| 0.211 \| \| (0.138) \| (0.200) \| (0.401) \| (0.430) \| \| *Only gives* \| 0.151*** \| 0.621*** \| 0.383*** \| 0.605*** \| \| (0.047) \| (0.116) \| (0.120) \| (0.196) \| \| *Both receives and gives* \| 0.201 \| 0.094 \| 0.559 \| 0.198 \| \| (0.161) \| (0.271) \| (0.359) \| (0.334) \| |

Note: *Pseudo R-squared (0.051); Number of observations (13,586); Other control variable included: ‘household size’, ‘having the partner in the same household’, ‘self-perceived health score, ‘activities of daily living (ADL) scale’, ‘having been hospitalized in the last 12 months’, ‘income quintiles’, ‘employment and retirement status’, and a list of dummies for the country of residence.; Standard deviations in brackets; * significant at 10%; ** significant at 5%; *** significant at*

**Table S3**. Selected coefficients of the multinomial regression model for selected variables – Wave 5 of SHARE data

| A3.A Age between 65 and 75 years old (ref: younger than 65) | A3.B Age older than 75 years old (ref: younger than 65) |
| --- | --- |
| \| Informal  care  Fin. gifts \| *Neither receives nor gives* \| *Only receives* \| *Only gives* \| *Both receives and gives* \| \| --- \| --- \| --- \| --- \| --- \| \| *Neither receives nor gives* \|  \| 0.110** \| -0.324*** \| -0.371*** \| \|  \| (0.055) \| (0.039) \| (0.064) \| \| *Only receives* \| -0.390*** \| 0.167 \| -0.837*** \| -0.703*** \| \| (0.097) \| (0.162) \| (0.132) \| (0.164) \| \| *Only gives* \| 0.012 \| 0.022 \| -0.259*** \| -0.484*** \| \| (0.039) \| (0.084) \| (0.048) \| (0.079) \| \| *Both receives and gives* \| -0.362*** \| 0.147 \| -0.728*** \| -0.574*** \| \| (0.094) \| (0.196) \| (0.104) \| (0.132) \| | \| Informal  care  Fin. gifts \| *Neither receives nor gives* \| *Only receives* \| *Only gives* \| *Both receives and gives* \| \| --- \| --- \| --- \| --- \| --- \| \| *Neither receives nor gives* \|  \| 0.845*** \| -0.965*** \| -0.639*** \| \|  \| (0.050) \| (0.051) \| (0.074) \| \| *Only receives* \| -0.655*** \| 0.400** \| -1.525*** \| -1.181*** \| \| (0.113) \| (0.156) \| (0.192) \| (0.205) \| \| *Only gives* \| -0.121*** \| 0.604*** \| -0.977*** \| -0.735*** \| \| (0.045) \| (0.080) \| (0.069) \| (0.098) \| \| *Both receives and gives* \| -0.121*** \| 0.604*** \| -0.977*** \| -0.735*** \| \| (0.045) \| (0.080) \| (0.069) \| (0.098) \| |
| **A3.C** Gender: female |  |
| \| Informal  care  Fin. gifts \| *Neither receives nor gives* \| *Only receives* \| *Only gives* \| *Both receives and gives* \| \| --- \| --- \| --- \| --- \| --- \| \| *Neither receives nor gives* \|  \| 0.434*** \| 0.185*** \| 0.272*** \| \|  \| (0.042) \| (0.034) \| (0.057) \| \| *Only receives* \| 0.233*** \| 0.703*** \| 0.606*** \| 0.846*** \| \| (0.085) \| (0.138) \| (0.117) \| (0.166) \| \| *Only gives* \| -0.158*** \| 0.246*** \| 0.134*** \| 0.164** \| \| (0.033) \| (0.065) \| (0.042) \| (0.068) \| \| *Both receives and gives* \| 0.179** \| 0.473*** \| 0.265*** \| 0.515*** \| \| (0.081) \| (0.162) \| (0.089) \| (0.122) \| |  |

Note: *Pseudo R-squared (0.084); Number of observations (41,030); Other control variable included: ‘household size’, ‘having the partner in the same household’, ‘self-perceived health score, ‘activities of daily living (ADL) scale’, ‘having been hospitalized in the last 12 months’, ‘income quintiles’, and a list of dummies for the country of residence.; Standard deviations in brackets; * significant at 10%; ** significant at 5%; *** significant at 1%*

**Table S4**. Selected coefficients of the multinomial regression model for the pooled data

| A4.A Age between 65 and 75 years old (ref: younger than 65) | A4.B Age older than 75 years old (ref: younger than 65) |
| --- | --- |
| \| Informal  care  Fin. gifts \| *Neither receives nor gives* \| *Only receives* \| *Only gives* \| *Both receives and gives* \| \| --- \| --- \| --- \| --- \| --- \| \| *Neither receives nor gives* \|  \| 0.243*** \| -0.366*** \| -0.223*** \| \|  \| (0.045) \| (0.034) \| (0.056) \| \| *Only receives* \| -0.282*** \| 0.196 \| -0.712*** \| -0.477*** \| \| (0.082) \| (0.132) \| (0.114) \| (0.151) \| \| *Only gives* \| 0.039 \| 0.235*** \| -0.317*** \| -0.301*** \| \| (0.034) \| (0.073) \| (0.044) \| (0.075) \| \| *Both receives and gives* \| -0.136 \| 0.280* \| -0.600*** \| -0.429*** \| \| (0.085) \| (0.164) \| (0.097) \| (0.121) \| | \| Informal  care  Fin. gifts \| *Neither receives nor gives* \| *Only receives* \| *Only gives* \| *Both receives and gives* \| \| --- \| --- \| --- \| --- \| --- \| \| *Neither receives nor gives* \|  \| 1.050*** \| -0.987*** \| -0.437*** \| \|  \| (0.042) \| (0.043) \| (0.063) \| \| *Only receives* \| -0.575*** \| 0.440*** \| -1.362*** \| -0.804*** \| \| (0.097) \| (0.132) \| (0.157) \| (0.180) \| \| *Only gives* \| -0.055 \| 0.839*** \| -1.035*** \| -0.407*** \| \| (0.039) \| (0.072) \| (0.061) \| (0.087) \| \| *Both receives and gives* \| -0.259** \| 0.631*** \| -1.280*** \| -0.788*** \| \| (0.102) \| (0.169) \| (0.145) \| (0.156) \| |
| A4.C Gender: female | A4.D Wave identifier: Wave 4 is the reference category |
| \| Informal  care  Fin. gifts \| *Neither receives nor gives* \| *Only receives* \| *Only gives* \| *Both receives and gives* \| \| --- \| --- \| --- \| --- \| --- \| \| *Neither receives nor gives* \|  \| 0.414*** \| 0.229*** \| 0.305*** \| \|  \| (0.031) \| (0.025) \| (0.042) \| \| *Only receives* \| 0.330*** \| 0.774*** \| 0.609*** \| 0.878*** \| \| (0.063) \| (0.109) \| (0.086) \| (0.128) \| \| *Only gives* \| -0.146*** \| 0.236*** \| 0.158*** \| 0.218*** \| \| (0.025) \| (0.049) \| (0.032) \| (0.052) \| \| *Both receives and gives* \| 0.186*** \| 0.640*** \| 0.379*** \| 0.466*** \| \| (0.062) \| (0.124) \| (0.069) \| (0.091) \| | \| Informal  care  Fin. gifts \| Neither receives nor gives \| Only receives \| Only gives \| Both receives and gives \| \| --- \| --- \| --- \| --- \| --- \| \| *Neither receives nor gives* \|  \| -0.013 \| 0.115*** \| 0.095 \| \|  \| (0.054) \| (0.042) \| (0.070) \| \| *Only receives* \| -0.065 \| 0.112 \| -0.125 \| 0.148 \| \| (0.096) \| (0.166) \| (0.125) \| (0.169) \| \| *Only gives* \| -0.047 \| 0.123 \| -0.022 \| -0.029 \| \| (0.046) \| (0.097) \| (0.057) \| (0.093) \| \| *Both receives and gives* \| 0.049 \| -0.386* \| 0.041 \| -0.111 \| \| (0.112) \| (0.211) \| (0.117) \| (0.154) \| |

Note: *Pseudo R-squared (0.082); Number of observations (76,393); Other control variable included: ‘household size’, ‘having the partner in the same household’, ‘self-perceived health score, ‘activities of daily living (ADL) scale’, ‘having been hospitalized in the last 12 months’, ‘income quintiles’, ‘employment and retirement status’, and a list of dummies for the country of residence.; Standard deviations in brackets; * significant at 10%; ** significant at 5%; *** significant at 1%*

**Table S5**. Coefficients of the multinomial regression model for the multinomial logistic model with random effects– Wave 4 and 5 of SHARE data

| A5.A Age between 65 and 75 years old (ref: younger than 65) | A5.B Age older than 75 years old (ref: younger than 65) |
| --- | --- |
| \| Informal  care  Fin. gifts \| *Neither receives nor gives* \| *Only receives* \| *Only gives* \| *Both receives and gives* \| \| --- \| --- \| --- \| --- \| --- \| \| *Neither receives nor gives* \|  \| 0.429*** \| -0.338*** \| -0.107** \| \|  \| (0.037) \| (0.027) \| (0.045) \| \| *Only receives* \| -0.298*** \| 0.408*** \| -0.776*** \| -0.467*** \| \| (0.068) \| (0.112) \| (0.095) \| (0.123) \| \| *Only gives* \| -0.083*** \| 0.367*** \| -0.437*** \| -0.440*** \| \| (0.027) \| (0.060) \| (0.034) \| (0.058) \| \| *Both receives and gives* \| -0.347*** \| 0.253* \| -0.825*** \| -0.541*** \| \| (0.068) \| (0.133) \| (0.079) \| (0.097) \| | \| Informal  care  Fin. gifts \| *Neither receives nor gives* \| *Only receives* \| *Only gives* \| *Both receives and gives* \| \| --- \| --- \| --- \| --- \| --- \| \| *Neither receives nor gives* \|  \| 1.484*** \| -1.058*** \| -0.294*** \| \|  \| (0.032) \| (0.036) \| (0.050) \| \| *Only receives* \| -0.578*** \| 0.890*** \| -1.504*** \| -0.842*** \| \| (0.079) \| (0.104) \| (0.136) \| (0.149) \| \| *Only gives* \| -0.366*** \| 1.014*** \| -1.405*** \| -0.722*** \| \| (0.031) \| (0.054) \| (0.051) \| (0.068) \| \| *Both receives and gives* \| -0.730*** \| 0.537*** \| -1.840*** \| -1.128*** \| \| (0.083) \| (0.128) \| (0.128) \| (0.129) \| |
| A5.C Gender: female |  |
| \| Informal  care  Fin. gifts \| *Neither receives nor gives* \| *Only receives* \| *Only gives* \| *Both receives and gives* \| \| --- \| --- \| --- \| --- \| --- \| \| *Neither receives nor gives* \|  \| 0.591*** \| 0.175*** \| 0.337*** \| \|  \| (0.028) \| (0.024) \| (0.041) \| \| *Only receives* \| 0.402*** \| 1.001*** \| 0.606*** \| 0.975*** \| \| (0.061) \| (0.104) \| (0.085) \| (0.124) \| \| *Only gives* \| -0.271*** \| 0.178*** \| 0.012 \| 0.110** \| \| (0.023) \| (0.046) \| (0.030) \| (0.050) \| \| *Both receives and gives* \| 0.117** \| 0.701*** \| 0.304*** \| 0.467*** \| \| (0.059) \| (0.120) \| (0.067) \| (0.088) \| |  |

Note: *No other control variables included; Standard deviations in brackets; * significant at 10%; ** significant at 5%; *** significant at 1%*

**Table S6.** Full results for the multinomial regression model for transfers from and to children

|  | *(1) Neither care & Neither financial* | *(2) Receives care & Neither financial* | *(3) Gives care & Neither financial* | *(4) Both care & Neither financial* | *(5) Neither care & Receives financial* | *(6) Receives care & Receives financial* | *(7) Gives care & Receives financial* | *(8) Both care & Receives financial* | *(9) Neither care & Gives financial* | *(10) Receives care & Gives financial* | *(11) Gives care & Gives financial* | *(12) Both care & Gives financial* | *(13) Neither care & Both financial* | *(14) Receives care & Both financial* | *(15) Gives care & Both financial* | *(16) Both care & both financial* |
| --- | --- | --- | --- | --- | --- | --- | --- | --- | --- | --- | --- | --- | --- | --- | --- | --- |
| Age 65-75 | *-* | 0.166** | -0.363*** | -0.220*** | -0.146 | 0.052 | -0.479*** | -0.373 | -0.024 | 0.262** | -0.461*** | -0.372*** | -0.049 | 0.406 | -0.637*** | -0.674*** |
|  |  | (0.068) | (0.050) | (0.085) | (0.122) | (0.202) | (0.174) | (0.252) | (0.049) | (0.117) | (0.066) | (0.121) | (0.130) | (0.248) | (0.156) | (0.186) |
| Age higher 75 | *-* | 0.934*** | -1.012*** | -0.546*** | -0.508*** | 0.106 | -1.129*** | -0.787*** | -0.083 | 0.804*** | -1.220*** | -0.469*** | -0.228 | 0.397 | -1.328*** | -1.128*** |
|  |  | (0.066) | (0.067) | (0.100) | (0.149) | (0.208) | (0.240) | (0.301) | (0.058) | (0.117) | (0.099) | (0.141) | (0.166) | (0.274) | (0.248) | (0.251) |
| Gender | *-* | 0.312*** | 0.235*** | 0.253*** | 0.431*** | 0.857*** | 0.613*** | 0.874*** | -0.098*** | 0.090 | 0.164*** | 0.183** | 0.237** | 0.667*** | 0.528*** | 0.301** |
|  |  | (0.049) | (0.039) | (0.067) | (0.097) | (0.183) | (0.130) | (0.206) | (0.037) | (0.082) | (0.049) | (0.085) | (0.096) | (0.203) | (0.109) | (0.139) |
| Partner in HH | *-* | -0.386*** | -0.046 | -0.508*** | -0.532*** | -0.585*** | -0.444*** | -1.025*** | 0.101** | -0.465*** | 0.097 | -0.693*** | -0.097 | -0.379* | -0.296** | -0.713*** |
|  |  | (0.057) | (0.048) | (0.080) | (0.107) | (0.191) | (0.145) | (0.220) | (0.047) | (0.099) | (0.065) | (0.107) | (0.121) | (0.226) | (0.132) | (0.170) |
| HH size | *-* | -0.155*** | 0.022 | -0.033 | 0.108*** | -0.178** | 0.134** | -0.045 | -0.065*** | -0.109** | -0.086*** | -0.035 | 0.061 | -0.124 | 0.091* | -0.028 |
|  |  | (0.028) | (0.021) | (0.038) | (0.040) | (0.090) | (0.056) | (0.094) | (0.021) | (0.050) | (0.029) | (0.049) | (0.047) | (0.110) | (0.050) | (0.077) |
| Self-perceived health | *-* | -0.392*** | 0.101*** | -0.058* | 0.007 | -0.435*** | 0.124** | -0.261*** | 0.102*** | -0.224*** | 0.128*** | 0.003 | 0.136*** | -0.176* | 0.210*** | -0.015 |
|  |  | (0.026) | (0.019) | (0.034) | (0.047) | (0.086) | (0.061) | (0.093) | (0.019) | (0.043) | (0.025) | (0.044) | (0.049) | (0.098) | (0.054) | (0.071) |
| In hospital in last 12 months | *-* | 0.475*** | -0.207*** | 0.300*** | 0.236** | 0.388** | -0.023 | 0.290 | 0.122** | 0.637*** | -0.195** | 0.308*** | -0.255* | 0.254 | -0.028 | 0.396** |
|  |  | (0.051) | (0.059) | (0.081) | (0.119) | (0.163) | (0.179) | (0.219) | (0.050) | (0.086) | (0.078) | (0.110) | (0.151) | (0.212) | (0.163) | (0.170) |
| Limitations with IADL | *-* | 0.349*** | -0.186*** | 0.205*** | 0.055 | 0.400*** | -0.107 | 0.162 | 0.008 | 0.353*** | -0.171*** | 0.236*** | 0.148* | 0.439*** | 0.020 | 0.026 |
|  |  | (0.021) | (0.044) | (0.041) | (0.067) | (0.056) | (0.137) | (0.117) | (0.033) | (0.034) | (0.065) | (0.058) | (0.080) | (0.075) | (0.124) | (0.128) |
| Employment - retired | *-* | 0.172** | 0.026 | 0.103 | -0.322*** | -0.156 | -0.353** | -0.971*** | -0.166*** | 0.003 | -0.175*** | -0.278** | -0.450*** | -0.556** | -0.496*** | -0.128 |
|  |  | (0.080) | (0.051) | (0.092) | (0.124) | (0.242) | (0.163) | (0.237) | (0.050) | (0.130) | (0.064) | (0.115) | (0.126) | (0.270) | (0.135) | (0.175) |
| Employment -unemployed | *-* | -0.097 | 0.077 | 0.080 | 0.252 | -0.209 | 0.669*** | -0.420 | -0.656*** | -1.007** | -0.482*** | -0.142 | -0.300 | -0.384 | -0.175 | -0.230 |
|  |  | (0.164) | (0.091) | (0.169) | (0.191) | (0.493) | (0.211) | (0.413) | (0.122) | (0.423) | (0.145) | (0.228) | (0.267) | (0.613) | (0.254) | (0.380) |
| Employment - disabled | *-* | 0.627*** | -0.147 | 0.153 | -0.093 | 0.364 | -0.078 | -0.581 | -0.628*** | -0.232 | -0.411*** | -0.194 | -0.981** | 0.238 | -0.707** | 0.396 |
|  |  | (0.112) | (0.105) | (0.155) | (0.221) | (0.326) | (0.300) | (0.402) | (0.126) | (0.236) | (0.150) | (0.216) | (0.395) | (0.422) | (0.354) | (0.305) |
| Employment - homemaker | *-* | 0.311*** | 0.110 | 0.143 | -0.204 | -0.209 | -0.077 | -0.815* | -0.442*** | 0.055 | -0.252** | -0.711*** | -0.794*** | -0.112 | -0.627** | -0.328 |
|  |  | (0.107) | (0.080) | (0.154) | (0.191) | (0.354) | (0.246) | (0.439) | (0.090) | (0.193) | (0.111) | (0.268) | (0.249) | (0.434) | (0.264) | (0.450) |
| Quintile 1 | *-* | -0.021 | -0.408*** | -0.540*** | -0.154 | 0.677 | -0.145 | -0.050 | -1.430*** | -1.530*** | -1.723*** | -2.200*** | -1.696*** | -0.805* | -1.914*** | -1.858*** |
|  |  | (0.109) | (0.082) | (0.148) | (0.192) | (0.452) | (0.255) | (0.433) | (0.081) | (0.178) | (0.116) | (0.198) | (0.214) | (0.439) | (0.243) | (0.327) |
| Quintile 2 | *-* | -0.012 | -0.212*** | -0.304** | -0.037 | 0.402 | 0.076 | -0.268 | -1.049*** | -1.297*** | -1.109*** | -1.425*** | -1.172*** | -0.425 | -1.182*** | -1.107*** |
|  |  | (0.099) | (0.073) | (0.131) | (0.175) | (0.432) | (0.227) | (0.409) | (0.070) | (0.155) | (0.096) | (0.165) | (0.183) | (0.388) | (0.205) | (0.280) |
| Quintile 3 | *-* | -0.084 | -0.138** | -0.326*** | -0.436** | 0.299 | -0.203 | 0.015 | -0.770*** | -0.853*** | -0.830*** | -1.129*** | -0.828*** | -0.688* | -0.916*** | -0.903*** |
|  |  | (0.095) | (0.064) | (0.119) | (0.173) | (0.425) | (0.213) | (0.372) | (0.060) | (0.134) | (0.080) | (0.142) | (0.159) | (0.380) | (0.179) | (0.255) |
| Quintile 4 | *-* | -0.087 | 0.034 | -0.056 | -0.278 | 0.371 | 0.000 | 0.301 | -0.245*** | -0.396*** | -0.236*** | -0.690*** | -0.248* | -0.183 | -0.238 | -0.469** |
|  |  | (0.098) | (0.059) | (0.113) | (0.170) | (0.436) | (0.195) | (0.357) | (0.053) | (0.128) | (0.067) | (0.128) | (0.139) | (0.363) | (0.149) | (0.238) |
| SN members <5km2 | *-* | 0.402*** | 0.497*** | 0.621*** | -0.050 | 0.419** | 0.200 | 0.222 | 0.127** | 0.593*** | 0.482*** | 0.538*** | 0.231* | -0.029 | 0.498*** | 0.615*** |
|  |  | (0.057) | (0.053) | (0.084) | (0.125) | (0.178) | (0.169) | (0.227) | (0.053) | (0.102) | (0.071) | (0.117) | (0.139) | (0.239) | (0.152) | (0.182) |
| *Belgium* | *-* | 0.178* | 0.999*** | 0.816*** | -0.251 | -0.409 | 0.286 | 0.346 | -0.319*** | 0.070 | 0.578*** | 0.033 | -0.967*** | -0.833** | -0.509** | -0.769** |
|  |  | (0.102) | (0.083) | (0.149) | (0.205) | (0.412) | (0.238) | (0.402) | (0.080) | (0.165) | (0.105) | (0.192) | (0.208) | (0.422) | (0.208) | (0.351) |
| *Denmark* | *-* | 0.795*** | 1.153*** | 2.074*** | -0.523 | 0.941** | 0.749** | 1.535*** | 0.026 | 0.886*** | 0.908*** | 1.643*** | -0.568** | 0.117 | 0.219 | 1.281*** |
|  |  | (0.140) | (0.112) | (0.162) | (0.382) | (0.457) | (0.313) | (0.434) | (0.108) | (0.201) | (0.133) | (0.186) | (0.274) | (0.511) | (0.241) | (0.290) |
| *Germany* | *-* | 0.072 | 0.612*** | 0.731*** | -0.208 | 0.369 | 0.387 | 0.522 | 0.243** | 0.634*** | 0.713*** | 0.796*** | -0.243 | -1.610 | -0.132 | 0.285 |
|  |  | (0.154) | (0.130) | (0.219) | (0.332) | (0.478) | (0.365) | (0.584) | (0.109) | (0.207) | (0.153) | (0.245) | (0.279) | (1.027) | (0.333) | (0.429) |
| *Netherlands* | *-* | -0.180 | 0.583*** | 0.757*** | -0.364 | -0.260 | -0.027 | 0.835* | -0.349*** | 0.071 | 0.435*** | 0.343 | -1.957*** | -1.162* | -0.694** | -2.571** |
|  |  | (0.139) | (0.102) | (0.176) | (0.267) | (0.511) | (0.315) | (0.429) | (0.097) | (0.204) | (0.123) | (0.215) | (0.396) | (0.623) | (0.273) | (1.021) |
| *Sweden* | *-* | 0.162 | 1.075*** | 1.109*** | -0.093 | -0.729 | 0.631* | 0.184 | 0.125 | 0.421** | 1.217*** | 0.733*** | -0.935*** | -0.112 | -0.634* | 0.450 |
|  |  | (0.145) | (0.112) | (0.191) | (0.311) | (0.750) | (0.333) | (0.653) | (0.103) | (0.207) | (0.126) | (0.230) | (0.326) | (0.508) | (0.362) | (0.377) |
| *Switzerland* | *-* | -0.240* | 0.291*** | -0.161 | -0.587** | -0.306 | -0.172 | -1.037 | -0.609*** | -0.703*** | 0.027 | -0.731*** | -1.053*** | -1.243** | -1.133*** | -1.574*** |
|  |  | (0.130) | (0.097) | (0.199) | (0.240) | (0.496) | (0.286) | (0.666) | (0.084) | (0.209) | (0.115) | (0.228) | (0.205) | (0.527) | (0.243) | (0.466) |
| *France* | *-* | -0.417*** | 0.554*** | -0.124 | -0.540*** | -1.286*** | -0.189 | -1.510** | -0.168** | -0.770*** | 0.188* | -0.500** | -1.559*** | -1.807*** | -1.313*** | -1.789*** |
|  |  | (0.103) | (0.084) | (0.170) | (0.205) | (0.473) | (0.251) | (0.649) | (0.072) | (0.191) | (0.107) | (0.211) | (0.240) | (0.548) | (0.258) | (0.488) |
| *Italy* | *-* | -0.131 | 0.224** | 0.018 | 0.168 | 0.787** | -0.408 | -0.097 | 0.256*** | 0.212 | 0.647*** | -0.462 | 0.059 | -1.476** | -0.281 | -0.649 |
|  |  | (0.118) | (0.105) | (0.204) | (0.206) | (0.322) | (0.333) | (0.546) | (0.083) | (0.185) | (0.121) | (0.296) | (0.187) | (0.627) | (0.250) | (0.458) |
| *Portugal* | *-* | -0.473*** | 0.017 | -0.117 | -0.603** | -1.162** | -1.209** | -0.308 | -0.318*** | -0.464* | -0.010 | -0.644* | -0.905*** | -0.690 | -0.930** | -1.997* |
|  |  | (0.141) | (0.126) | (0.238) | (0.281) | (0.571) | (0.491) | (0.601) | (0.117) | (0.274) | (0.175) | (0.385) | (0.329) | (0.523) | (0.407) | (1.024) |
| *Spain* | *-* | -0.419*** | -0.319*** | -0.643*** | -0.707*** | -1.371*** | -1.072*** | -1.506* | -0.656*** | -1.512*** | -0.829*** | -1.682*** | -1.692*** | -3.123*** | -2.135*** | -1.663*** |
|  |  | (0.113) | (0.110) | (0.224) | (0.237) | (0.521) | (0.356) | (0.777) | (0.100) | (0.299) | (0.175) | (0.435) | (0.327) | (1.036) | (0.470) | (0.617) |
| *Czech Rep.* | *-* | 0.538*** | 0.503*** | 1.556*** | 0.188 | 1.187*** | 0.151 | 1.768*** | 0.354*** | 0.967*** | 0.874*** | 1.725*** | 0.704*** | 1.369*** | 0.803*** | 2.154*** |
|  |  | (0.100) | (0.095) | (0.149) | (0.190) | (0.300) | (0.261) | (0.361) | (0.084) | (0.173) | (0.121) | (0.183) | (0.178) | (0.314) | (0.203) | (0.254) |
| *Estonia* | *-* | 0.111 | 0.586*** | 0.778*** | 0.054 | -0.425 | -0.079 | -0.600 | 0.307*** | 0.545*** | 0.719*** | 0.920*** | 0.135 | -0.361 | 0.643*** | 0.508 |
|  |  | (0.100) | (0.093) | (0.159) | (0.188) | (0.340) | (0.266) | (0.471) | (0.084) | (0.176) | (0.125) | (0.203) | (0.198) | (0.378) | (0.212) | (0.315) |
| *Hungary* | *-* | -0.230* | 0.353*** | 0.459** | -0.378 | -0.361 | -0.678* | -0.251 | 0.080 | 0.002 | 0.381** | 0.628** | -0.382 | -1.741** | -0.322 | -1.049* |
|  |  | (0.122) | (0.110) | (0.189) | (0.240) | (0.392) | (0.368) | (0.539) | (0.106) | (0.237) | (0.160) | (0.253) | (0.286) | (0.768) | (0.336) | (0.628) |
| *Poland* | *-* | -0.344** | 0.061 | -0.471 | 0.086 | -0.208 | 0.054 | 0.245 | 0.527*** | 0.259 | 0.931*** | 0.111 | 0.161 | -1.198 | 0.413 | -0.696 |
|  |  | (0.152) | (0.148) | (0.316) | (0.253) | (0.453) | (0.362) | (0.584) | (0.121) | (0.273) | (0.180) | (0.400) | (0.312) | (0.782) | (0.349) | (0.758) |
| *Slovenia* | *-* | -0.654*** | -0.172 | -0.842*** | -0.679*** | -2.123*** | -1.067*** | -1.359* | -0.438*** | -0.715*** | -0.301** | -0.633** | -1.000*** | -2.022*** | -1.059*** | -1.388** |
|  |  | (0.126) | (0.108) | (0.247) | (0.239) | (0.753) | (0.379) | (0.772) | (0.091) | (0.233) | (0.143) | (0.275) | (0.243) | (0.747) | (0.299) | (0.539) |
| Constant | *-* | -1.321*** | -1.995*** | -2.742*** | -2.910*** | -4.003*** | -4.158*** | -3.570*** | -0.608*** | -2.004*** | -1.720*** | -2.017*** | -2.541*** | -3.456*** | -3.092*** | -2.944*** |
|  |  | (0.162) | (0.126) | (0.220) | (0.288) | (0.597) | (0.379) | (0.601) | (0.114) | (0.257) | (0.157) | (0.266) | (0.287) | (0.592) | (0.318) | (0.427) |
| *N* |  | 35,363 | 35,363 | 35,363 | 35,363 | 35,363 | 35,363 | 35,363 | 35,363 | 35,363 | 35,363 | 35,363 | 35,363 | 35,363 | 35,363 | 35,363 |
|  |  |  |  |  |  |  |  |  |  |  |  |  |  |  |  |  |

*Pseudo R-squared (0.114); Standard deviations in brackets; * significant at 10%; ** significant at 5%; *** significant at 1%*
